# Perioperative complications of focal therapy for prostate cancer: results from the GeRmAn Nationwide inpatient Data (GRAND) study

**DOI:** 10.1111/bju.16746

**Published:** 2025-04-19

**Authors:** Nikolaos Pyrgidis, Michael Chaloupka, Benedikt Ebner, Yannic Volz, Philipp Weinhold, Julian Marcon, Lennert Eismann, Christian G. Stief, Gerald B. Schulz, Maria Apfelbeck

**Affiliations:** ^1^ Department of Urology LMU Klinikum Munich Germany

**Keywords:** complications, epidemiological study, focal therapy, HIFU, prostate cancer

## Abstract

**Objective:**

To compare the perioperative complications of the most common focal therapy (FT) modalities for prostate cancer.

**Patients and Methods:**

We assessed the GeRmAn Nationwide inpatient Data (GRAND) from 2005 to 2023, provided by the Research Data Center of the Federal Bureau of Statistics. We compared perioperative outcomes of high‐intensity focused ultrasound (HIFU), hyperthermia, irreversible electroporation of the prostate, cryotherapy, vascular photodynamic therapy of the prostate (VTP), and transurethral ultrasound ablation, as well as HIFU vs non‐HIFU treatments in general. Furthermore, we evaluated the role of concomitant transurethral resection of the prostate (TURP) on perioperative complications. Finally, the complication rate of FT was also compared to brachytherapy and robot‐assisted radical prostatectomy.

**Results:**

A total of 10 544 underwent FT. Most patients received HIFU (92%). The number of FT cases performed annually has been steadily decreasing. The most prevalent complication (9.6%) was urinary tract infection (HIFU: 10%, hyperthermia: 6.2%, cryotherapy: 6.8%, VTP: 3.9%). Haematuria was observed in 3.6% of all cases. In the multivariable regression, HIFU was associated with higher rates of urinary tract infections (10% vs 5.2%, *P* < 0.001) but lower rates of haematuria (3.4% vs 5.5%, *P* < 0.001) and admission to the intensive care unit (0.7% vs 2.2%, *P* < 0.001) compared to non‐HIFU procedures. Concomitant TURP was associated with higher transfusion (*P* < 0.001), haematuria (*P* < 0.001), sepsis (*P* = 0.001), and urinary retention rates (*P* = 0.03). Most perioperative complications were statistically significantly worse in patients undergoing FT compared to brachytherapy, while most perioperative complications were better after FT vs robot‐assisted radical prostatectomy.

**Conclusions:**

In the largest epidemiological comparative study on the risk of complications of the most common FT for prostate cancer, we were able to show an overall low risk of perioperative complications.

**Abbreviations**
FT: focal therapy
GRAND: GeRmAn Nationwide Inpatient Data
HIFU: high‐intensity focused ultrasound
ICD‐10: International Statistical Classification of Diseases and Related Health Problems 10th Revision
ICU: intensive care unit
IRE: irreversible electroporation of the prostate
IQR: interquartile range
ISUP: International Society of Urological Pathology
OPS: Operationen‐ und Prozedurenschlüssel
OR: odds ratio
PCa: prostate cancer
(RA)RP: (robot‐assisted) radical prostatectomy
TULSA: transurethral ultrasound ablation
VTP: vascular photodynamic therapy of the prostate

## Introduction

Focal therapy (FT) has emerged as a treatment option between active surveillance and definitive treatment of prostate cancer (PCa). FT usually treats only the tumorous part of the prostate, thus preserving continence and erectile function in most cases, while offering the chance of healing or postponement of definitive treatment [1, 2, 3, 4]. Epidemiological data suggest that the most common form of FT in Germany is high‐intensity focused ultrasound (HIFU) [5]. Apart from HIFU, other forms of FT are also regularly used and include hyperthermia, irreversible electroporation of the prostate (IRE), cryotherapy, vascular photodynamic therapy of the prostate (VTP) or transurethral ultrasound ablation (TULSA).

In 2021, FT was included in the German guidelines as a weak, non‐evidence‐based expert recommendation [6] only for patients with low‐risk PCa and a PSA value <10 ng/mL if they refuse definitive therapy or active surveillance [6]. Similarly, the European Guidelines for PCa do not recommend FT for patients with intermediate‐risk PCa [7, 8]. On the contrary, to the FocAL therapy CONsensus (FALCON) of 2024, which recommend that FT should be offered to patients with localised International Society of Urological Pathology (ISUP) Grade 2 (percentage of pattern 4 >10%) or ISUP Grade 3 PCa [9]. Furthermore, guidelines do not consider any form of FT more favourable regarding postoperative health‐related quality of life or oncological outcomes. Due to the lack of long‐term oncological data, the European Association of Urology guidelines suggest that FT, in the form of HIFU or cryotherapy, can be performed in the context of prospective registries and all other forms of FT should only be performed in the context of well‐designed prospective trials with a ‘strong’ recommendation [8].

To complicate things further, the existing studies evaluating the oncological outcomes of FT report contradictory findings [4, 10, 11, 12]. However, it should be noted that only a limited number of randomised controlled trials or high‐quality prospective studies on the matter exist [11, 13]. In light of the sparse data for oncological outcomes, the risk of adverse events and impact on health‐related quality of life is of particular significance. To date, no study has evaluated the comparative efficacy and safety of different modalities of FT in terms of perioperative outcomes and side effects. In this context, we present, to the best of our knowledge, the largest study on this topic to date assessing the current trends and major perioperative complications of different FT forms in Germany from 2005 until 2023. We have also compared FT vs brachytherapy and vs robot‐assisted radical prostatectomy (RARP) as standard therapies of PCa.

## Patients and Methods

### GeRmAn Nationwide Inpatient Data (GRAND)

The GRAND is a nationwide dataset that contains in‐hospital data from all patients in Germany between 2005 and 2023, except for those admitted for psychiatric, forensic, or military reasons. After 2005, all hospitals in Germany had to transfer relevant in‐hospital data to the German Institute for the Hospital Remuneration System to receive their remuneration. These data are stored anonymised and coded at the Research Data Center of the Federal Bureau of Statistics. They are coded based on the International Statistical Classification of Diseases and Related Health Problems, 10th revision, German modification (ICD‐10‐GM), and the German Procedure Classification [4] and contain information on baseline diagnoses, in‐hospital procedures, and perioperative outcomes. These data capture only the inpatient post‐procedure hospital stay and no information is available after the patient is discharged. The corresponding coding guidelines are provided by the German Institute for Medical Documentation and Information and ensure uniform documentation throughout Germany. Subsequently, these data are evaluated by independent physician task forces from healthcare insurance companies and display a high degree of accuracy.

Our research team did not have direct access to these data but could access their summary results after an agreement between our department and the Research Data Center of the Federal Bureau of Statistics (LMU – 4710‐2022). All analyses were undertaken on our behalf, from the Research Data Center based on R codes provided by our research team. Subsequently, the Research Data Center of the Federal Bureau of Statistics sent to our research team the summary findings for further assessment (source: Research Data Center of the Federal Bureau of Statistics, DRG Statistics 2005–2023, own calculations). Following German legislation, approval by an ethics committee or patient informed consent was not mandatory for the present analysis from the GRAND study.

### Outcomes

We included all hospitalised patients undergoing FT for PCa (ICD‐10 code: C61) in Germany. More specifically, we included patients undergoing HIFU (Operationen‐ und Prozedurenschlüssel [OPS] code: 5‐602.1), hyperthermia (OPS code: 5‐602.0), IRE (OPS code: 5‐602.6), cryotherapy (OPS code: 5‐602.3), VTP (OPS code: 5‐602.5) and TULSA (OPS code: 5‐601.a). Moreover, we included patients undergoing brachytherapy (OPS code: 8‐524 or 8‐525) and RARP (OPS code: 5‐604 and 5‐987) for PCa. The primary outcome of the study was to assess perioperative in‐hospital mortality, admission to the intensive care unit (ICU), morbidity (UTI [ICD‐10 code: N39.0], urinary retention [ICD‐10 code: R33], haematuria [ICD‐10 code: R31], acute kidney disease [ICD‐10 code: N17], sepsis [ICD‐10 code: A41], transfusion [OPS code: 8‐80], and need for colonoscopy [OPS code: 1‐65]), as well as the length of hospital stay of the six available FT modalities for PCa. Secondary outcomes included the role of concomitant FT with TURP (OPS code: 5‐601.1 or 5‐601.0) on perioperative outcomes, as well as the comparison of FT vs brachytherapy and vs RARP.

### Statistical Analysis

All continuous variables were reported as median with interquartile range (IQR) and all categorical variables as frequencies with proportions. When less than three cases of an outcome were reported, these data were censored by the German Federal Statistical Office to prevent individual patient identification. We undertook a multivariable logistic and linear regression analysis to assess the perioperative outcomes after FT for PCa (HIFU vs non‐HIFU modalities), the concomitant treatment with TURP and FT vs brachytherapy and vs RARP. Based on clinical relevance, all models were adjusted for age, diabetes, hypertension, obesity, chronic kidney disease, and BPH. Odds ratios (ORs) with 95% CIs [14] were provided for all logistic models and a two‐sided *P* < 0.05 was considered statistically significant.

## Results

### Baseline Characteristics and Perioperative Outcomes of FT Modalities

Patient demographics, comorbidities, distribution, perioperative outcomes, and major complications of FT modalities are listed in Table 1. A total of 10 544 patients underwent FT for PCa from 2005 to 2023. Most patients underwent HIFU (9703 [92%]). Furthermore, 130 (1%) patients underwent hyperthermia, 26 (0.2%) IRE, 339 (3%) cryotherapy, 180 (2%) VTP, and 166 (2%) TULSA. The median (IQR) patient age of the whole study population was 72 (66–76) years. Concerning comorbidities, 629 (6%) patients had BPH, 409 (3.9%) had urinary incontinence, and 5151 (49%) had hypertension.

**Table 1:** Baseline characteristics, perioperative outcomes, and major complications of patients undergoing FT for PCa.

Characteristic	Overall, *N* = 10 544	HIFU, *n* = 9703	Hyperthermia, *n* = 130	IRE, *n* = 26	Cryotherapy, *n* = 339	VTP, *n* = 180	TULSA, *n* = 166
Age, years, median (IQR)	72 (66–76)	72 (67–76)	72 (66–77)	65 (60–70)	72 (67–76)	65 (59–71)	66 (60–72)
BPH, *n* (%)	629 (6)	592 (6.1)	8 (6.2)	4 (15)	13 (3.8)	5 (2.8)	7 (4.2)
Incontinence, *n* (%)	409 (3.9)	369 (3.8)	7 (5.4)	XXX	25 (7.4)	XXX	6 (3.6)
Erectile dysfunction, *n* (%)	25 (0.2)	XXX	XXX	XXX	XXX	0 (0)	0 (0)
Diabetes, *n* (%)	1267 (12)	1180 (12)	16 (12)	0 (0)	46 (14)	15 (8.3)	10 (6)
Chronic heart failure, *n* (%)	283 (2.7)	269 (2.8)	3 (2.3)	0 (0)	8 (2.4)	XXX	XXX
COPD, *n* (%)	563 (5.3)	534 (5.5)	4 (3.1)	XXX	14 (4.1)	XXX	6 (3.6)
CKD, *n* (%)	528 (5)	460 (4.7)	12 (9.2)	0 (0)	46 (14)	4 (2.2)	6 (3.6)
CVD, *n* (%)	172 (1.6)	155 (1.6)	8 (6.2)	0 (0)	6 (1.8)	XXX	XXX
Dementia, *n* (%)	36 (0.3)	32 (0.3)	XXX	0 (0)	XXX	0 (0)	0 (0)
Hypertension, *n* (%)	5151 (49)	4798 (49)	63 (48)	10 (38)	133 (39)	77 (43)	70 (42)
Obesity, *n* (%)	538 (5.1)	503 (5.2)	6 (4.6)	0 (0)	16 (4.7)	8 (4.4)	5 (3)
Hospital stay, days, median (IQR)	4 (3–6)	4 (3–6)	5 (3–7)	2 (1–2)	6 (4–7)	3 (2–4)	3 (2–3)
Hospital revenues, euros, median (IQR)	3631 (3474–3817)	3637 (3448–3780)	3525 (3420–3831)	3491 (1408–3491)	3684 (3578–3943)	3605 (3492–3984)	3608 (3491–3719)
Acute kidney disease, *n* (%)	30 (0.3)	22 (0.2)	XXX	0 (0)	5 (1.5)	0 (0)	XXX
UTI, *n* (%)	1013 (9.6)	969 (10)	8 (6.2)	XXX	23 (6.8)	7 (3.9)	XXX
Urinary retention, *n* (%)	592 (5.6)	535 (5.5)	8 (6.2)	XXX	43 (13)	XXX	3 (1.8)
Haematuria, *n* (%)	377 (3.6)	331 (3.4)	7 (5.4)	XXX	22 (6.5)	XXX	14 (8.4)
Sepsis, *n* (%)	20 (0.2)	20 (0.2)	0 (0)	0 (0)	0 (0)	0 (0)	0 (0)
Transfusion, *n* (%)	73 (0.7)	66 (0.7)	XXX	0 (0)	XXX	0 (0)	0 (0)
Admission to ICU, *n* (%)	86 (0.8)	68 (0.7)	XXX	0 (0)	13 (3.8)	XXX	XXX
Colonoscopy, *n* (%)	418 (4)	403 (4.2)	10 (7.7)	0 (0)	XXX	0 (0)	XXX
TURP, *n* (%)	862 (8.2)	839 (8.6)	19 (15)	0 (0)	4 (1.2)	0 (0)	0 (0)
In‐hospital mortality, *n* (%)	3 (<0.1)	XXX	XXX	0 (0)	0 (0)	0 (0)	0 (0)

Censorship due to data protection are marked as XXX.

CKD, chronic kidney disease; COPD, chronic obstructive pulmonary disease; CVD, cerebrovascular disease.

Overall, the median (IQR) hospital stay was 4 (3–6) days. The median (IQR) hospital stay for patients treated with HIFU was 4 (3–6) days, for hyperthermia 5 (3–7) days, for IRE 2 (1–2) days, for cryotherapy 6 (4–7) days, for VTP 3 (2–4) days, and for TULSA 3 (2–3) days. A UTI occurred in 1011 (9.8%) cases (HIFU: 10%, hyperthermia: 6.2%, cryotherapy: 6.8%, VTP: 3.9%). Accordingly, less than three cases of UTIs were reported in patients treated with IRE or TULSA. During hospital stay, 418 (4%) colonoscopies were performed.

**Fig. 1:**
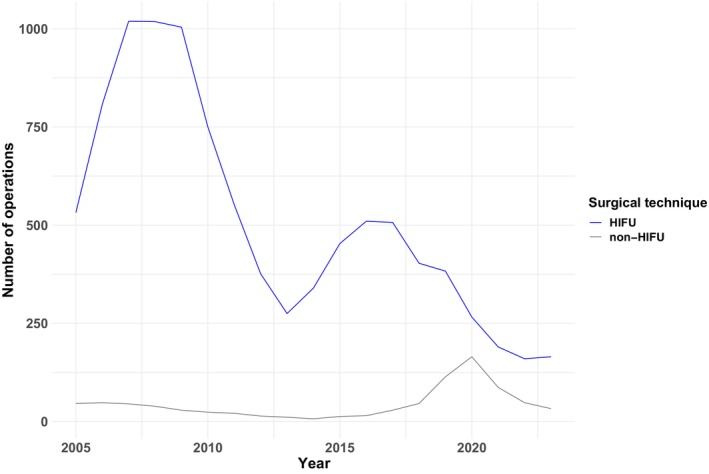
Annual trends of HIFU and non‐HIFU FT.

Figure 1 shows the annual trends of HIFU vs non‐HIFU modalities. Overall, the number of FT cases performed annually has been steadily decreasing. In 2007, 1064 cases (1019 HIFU and 45 non‐HIFU) were performed; in 2017, 536 cases (507 HIFU and 29 non‐HIFU) were performed. By 2023, only 198 patients (165 HIFU and 33 non‐HIFU) underwent FT. Furthermore, most patients (5715/10 544 [54%]) undergoing FT were aged between 70 and 79 years (HIFU: 5364/9703 [55%], non‐HIFU: 351/841 [42%]), followed by those aged 60–69 years (HIFU: 2671/9703 [28%], non‐HIFU: 287/841 [34%]).

### Comparison of Perioperative Outcomes: HIFU vs Non‐HIFU Modalities

The analysis comparing the complications of the FT modalities was summarised as HIFU vs non‐HIFU due to the small number of non‐HIFU cases (Table 2). In the multivariable regression analysis adjusted for age, diabetes, hypertension, obesity, chronic kidney disease, and BPH, UTIs occurred significantly more often after HIFU procedures compared to non‐HIFU procedures (10% vs 5.2%; OR 0.52, 95% CI 0.38–0.71; *P* < 0.001). On the contrary, haematuria (3.4% vs 5.5%; OR 1.8, 95% CI 1.3–2.4; *P* < 0.001) and admission to the ICU (0.7% vs 2.2%; OR 3.3, 95% CI 1.9–5.5; *P* < 0.001) occurred significantly less often after HIFU procedures compared to non‐HIFU procedures. The total number of sepsis, transfusion, and in‐hospital mortality rates were low and did not differ between HIFU and non‐HIFU procedures.

**Table 2:** Multivariable linear and logistic regression analysis for perioperative complications after FT for PCa (HIFU vs non‐HIFU).

Outcome	HIFU, *n* = 9520		Non‐HIFU, *n* = 840	
	Cases	Estimate (95% CI), *P*	Cases	Estimate (95% CI), *P*
UTI, *n* (%)	969 (10)	–	44 (5.3)	**OR 0.52 (0.38, 0.71), <0.001**
Haematuria, *n* (%)	331 (3.4)	–	46 (5.5)	**OR 1.8 (1.3, 2.4), <0.001**
Sepsis, *n* (%)	20 (0.2)	–	0 (0)	Not available, >0.9
Transfusion, *n* (%)	66 (0.7)	–	7 (0.8)	OR 1.3 (0.53, 2.7), 0.5
Admission to ICU, *n* (%)	68 (0.7)	–	18 (2.2)	**OR 3.3 (1.9, 5.5), <0.001**
Urinary retention, *n* (%)	535 (5.5)	–	57 (6.8)	OR 1.3 (0.95, 1.7), 0.1
Hospital stay, days, median (IQR)	4 (3–6)	–	3 (2–6)	Days −0.11 (−0.34, 0.12), 0.3

All models are adjusted for age, diabetes, hypertension, obesity, chronic kidney disease, and BPH. The bold cells indicate statistically significant *P* values.

### Comparison of Perioperative Outcomes: FT With or without Concomitant TURP

A TURP was only performed in cases where the patient underwent one of the following treatments: HIFU (839 patients), hyperthermia (19) or cryotherapy (four). In the multivariable regression analysis, concomitant TURP was associated with higher transfusion (OR 3.7, 95% CI 2.1–6.5; *P* < 0.001), haematuria (OR 2.6, 95% CI 1.9–3.4; *P* < 0.001), sepsis (OR 4.3, 95% CI 1.4–11.2; *P* = 0.001), and urinary retention rates (OR 1.4, 95% CI 1.03–1.8; *P* = 0.03), as well as with longer length of hospital stay (days difference: 3.4 [95% CI 3.1–3.6] days; *P* < 0.001) compared to no TURP. On the other hand, UTIs (OR 0.95, 95% CI 0.7–1.2; *P* = 0.7), admissions to the ICU (OR 1.3, 95% CI 0.6–2.5; *P* = 0.5) and in‐hospital mortality (no in‐hospital mortality cases) did not differ in patients undergoing FT with or without concomitant TURP.

### Comparison of Perioperative Outcomes: FT vs Brachytherapy and vs RARP

From 2005 to 2023, 10 544 patients underwent FT, 54 114 underwent brachytherapy, and 164 356 underwent RARP in Germany. The median (IQR) patient age was 70 (65–74) years for brachytherapy and 66 (61–71) years for RARP. The perioperative outcomes of FT vs brachytherapy and vs RARP are depicted in Table 3. In the multivariable regression analysis, apart from in‐hospital mortality and haematuria, all other perioperative complications were statistically significantly worse in patients undergoing FT compared to brachytherapy. On the contrary, apart from UTIs, all other perioperative complications were statistically significantly better in patients undergoing FT compared to RARP.

**Table 3:** Multivariable linear and logistic regression analysis for perioperative complications after FT vs brachytherapy and vs RARP for PCa.

Outcome	FT, *n* = 10 544		Brachytherapy, *n* = 54 114		RARP, *n* = 164 356	
	Cases	OR (95% CI), *P*	Cases	Estimate (95% CI), *P*	Cases	Estimate (95% CI), *P*
UTI, *n* (%)	1013 (9.6)	–	673 (1.2)	**OR 0.13 (0.12, 0.14), <0.001**	13 418 (8.2)	OR 0.97 (0.91, 1.04), 0.4
Haematuria, *n* (%)	377 (3.6)	–	3572 (6.6)	**OR 2.1 (1.9, 2**.**4), <0.001**	8241 (5)	**OR 1.6 (1.5, 1.8), <0.001**
Sepsis, *n* (%)	20 (0.2)	–	24 (<0.1)	**OR 0.25 (0.14, 0.46), <0.001**	535 (0.3)	**OR 1.8 (1.2, 2.9), 0.009**
Transfusion, *n* (%)	73 (0.7)	–	104 (0.2)	**OR 0.28 (0.21, 0.38), <0.001**	3874 (2.4)	**OR 3.5 (2.8, 4.5), <0.001**
In‐hospital mortality, *n* (%)	3 (<0.1)	–	12 (<0.1)	OR 0.81 (0.26, 3.6), 0.7	242 (0.1)	**OR 5.7 (2.2, 23), 0.003**
Admission to ICU, *n* (%)	86 (0.8)	–	122 (0.2)	**OR 0.28 (0.22, 0.38), <0.001**	8886 (5.4)	**OR 7 (5.7, 8.7), <0.001**
Urinary retention, *n* (%)	592 (5.6)	–	652 (1.2)	**OR 0.22 (0.2, 0.25), <0.001**	2399 (1.5)	**OR 0.28 (0.25, 0.3), <0.001**
Acute kidney disease, *n* (%)	30 (0.3)	–	31 (<0.1)	**OR 0.23 (0.14, 0.39), <0.001**	2594 (1.6)	**OR 6.9 (4.9, 10), <0.001**
Hospital stay, days, median (IQR)	4 (3–6)	–	2 (1–3)	Days **−2.5 (−2.6, −2.3), <0.001**	7 (6–8)	Days: **3 (2.9, 3.2), <0.001**

All models are adjusted for age, diabetes, hypertension, obesity, chronic kidney disease, and BPH. The bold cells indicate statistically significant *P* values.

## Discussion

Although several techniques exist for FT of PCa, data comparing the oncological and functional outcomes of these different modalities of FT for PCa are scarce. The existing evidence is predominantly based on retrospective cohort studies and case series. To the best of our knowledge, we present the first large‐scale study comparing the perioperative outcomes of different modalities of FT for the last 19 years. Moreover, we compared FT vs brachytherapy and vs RARP. The findings of the present study demonstrate that the overall rate of perioperative complications after FT is relatively low. In particular, UTIs occurred in 9.6% of treated patients, and haematuria occurred in 3.6% of treated patients. Based on the previous notion, severe complications were rare: blood transfusions occurred in 0.7% of all patients, admissions to the ICU in 0.8% of patients, and three patients (<0.1%) died during hospital stay for FT over the past 19 years.

Comparing HIFU as the most common form of FT to non‐HIFU modalities, we suggest that UTIs may occur more often after HIFU procedures than non‐HIFU procedures. On the contrary, haematuria may occur more often after non‐HIFU procedures than HIFU procedures. Of note, the rate of urinary retentions postoperatively and the overall hospital stay were similar between HIFU and non‐HIFU procedures. Interestingly, the concurrent performance of TURP increased the risk of postoperative urinary retention, transfusion, and haematuria after adjusting for the major baseline characteristics of the included patients. Importantly, most perioperative complications were statistically significantly worse in patients undergoing FT compared to brachytherapy, while most perioperative complications were better after FT vs RARP.

In recent years, FT for localised PCa has gained interest due to the recognition that low‐risk PCa is frequently subjected to overtreatment by RP or external beam radiotherapy [15]. However, FT is only recommended within the context of clinical trials or prospective registries for well‐selected patients [8], as long‐term high‐quality oncological data are lacking [16], and the existing studies on oncological outcomes are predominantly retrospective, exhibiting considerable heterogeneity in their study design, selection criteria, and comparator. The latter is also reflected in our analyses, which demonstrate that the number of annual FT cases has been steadily decreasing in Germany. This is supported by a recent analysis of the German hospital billing database by Flegar et al. [5] who evaluated current trends in different FT modalities. After consultation with the corresponding author, the discrepancy in the reported FT cases compared to our study is most likely due to the additional evaluation of the FT modality with the OPS codes 5‐602.y ‘various’ by Flegar et al. [5]. Moreover, a significant number of patients undergoing FT may require additional treatment within a short period of follow‐up [12, 17]. Therefore, it is not only mandatory to closely monitor these patients but also to minimise the short‐ and long‐term complications of FT.

The findings of the present study highlight that the rate of perioperative complications after FT is low. Available studies showed a large heterogeneity regarding postoperative complications of each type of FT. Still, it seems that most complications occur during the perioperative period [18]. Previous evidence suggests that postoperative UTI rates after HIFU range between 0.8% and 26.5% [19]. Accordingly, acute urinary retention is reported between 0% and 17% after primary and salvage HIFU therapies [20]. The findings of our study are in line with these studies, indicating that UTIs and acute urinary retentions may occur in 10% and 5.5% of all HIFU cases, respectively. A Cochrane analysis by Shelley et al. [21] reported urinary retention rates between 3% and 13% after cryotherapy. The findings of our study indicate that the prevalence of postoperative urinary retention may have been underestimated, as we report acute urinary retention rates of 13% following cryotherapy. Concerning VTP, the randomised controlled phase III trial by Azzouzi et al. [11] reported UTIs in 10% of patients, haematuria in 28% of patients, and urinary retention in 15% of patients after VTP therapy. Our analysis shows a lower occurrence of these complications.

Rectal–urethral fistulae are a rare complication of FT. Studies report occurrences of 0.1–0.4% after HIFU [18, 22]. As an indicator of potential rectal injury, we assessed the performance of colonoscopy after FT. A high number of postoperative colonoscopies was observed (4%), which may suggest a considerable discrepancy between the presumed rectal injury and the actual fistula. Of note, 7.7% of patients receiving hyperthermia underwent a colonoscopy during their hospital stay. However, it is important to note that this is an observation, and no causal relationship can be established based on the data. Importantly, we provide the first study assessing further perioperative complications such as blood transfusion and sepsis after FT and report very low rates.

It should be highlighted that our findings suggest higher rates of urinary retention, transfusion, and haematuria in patients undergoing concomitant TURP. Although our data cannot establish a causal relationship, it seems that this observation is due to the concomitant TURP, as TURP is associated with a perioperative transfusion rate of up to 7% [23]. This may question the prevailing practice of performing TURP before FT to reduce the risk of urinary tract complications [24]. Nevertheless, our findings are further supported by the comparative study of Baumunk et al. [25], who could not show a difference in micturition between the single or the combined approach. Nevertheless, other studies indicate that the combined approach may be associated with a reduced incidence of urinary retention and shorter catheter indwelling time, rendering the interpretation of our findings problematic [26, 27, 28]. Still, it should be stressed that our holistic approach combined with its large population may provide the best evidence on this matter.

In general, complication rates of FT depend on its indication. Studies evaluating whole‐gland treatment report higher rates of complications compared to FT [29]. Complication rates of salvage FT may be even higher but generally depending on the type of initial treatment. Studies evaluating the complication rates of primary vs salvage HIFU or cryotherapy indicate similar outcomes [30]. However, the complication rates of salvage FT after external beam radiation are higher compared to primary FT [18]. Of note, our findings demonstrate lower perioperative complications after brachytherapy compared to FT. As expected, patients undergoing RARP presented worse perioperative complications compared to FT or brachytherapy [31].

It should be noted that our study is subject to some limitations. One of the most significant limitations of our study is the considerable heterogeneity of our study population. We cannot provide information on whether FT was performed as a whole‐gland, salvage, or focal procedure. This is because the code used to document the procedure in Germany is identical for all three types of treatment. The high average age of the HIFU‐treated cohort may be attributed to a significant number of palliative whole‐gland treatments in the early years when HIFU was predominantly employed for patients unsuitable for RP [21]. This could also have a detrimental effect on our findings regarding postoperative complications following all types of FT. Importantly, given that, in Germany, radiation therapy for PCa is performed in an ambulatory setting and considering that we only had access to hospitalised cases, we could not compare FT to radiation therapy. Furthermore, reimbursement in the German healthcare system is tied to a minimum length of stay, which limits statements and comparisons about the length of hospital stay. In particular, the minimum length of stay for all surgical treatments for PCa, including FT and brachytherapy, is 2 days. Moreover, our analyses were derived from retrospective billing data that are prone to coding errors and misclassifications. Based on the previous notion, important patient information such as laboratory values (including PSA) and the oncological status (i.e., histology findings, ISUP Grade, tumour size and location, and TNM classification) is not collected. Similarly, data on tumour recurrence or progression, MRI findings, additional prostate biopsies or complications after hospital discharge, and functional outcomes in terms of urinary continence and sexual function are not available. Over the study period, the field of FT showed a high level of innovation with the emergence of different treatment modalities. Therefore, when comparing the established HIFU procedure with non‐HIFU procedures, it cannot be ruled out that perioperative complications may occur due to an existing learning curve. It should be also acknowledged that the FT groups were mismatched in size, which might have reduced the statistical power of our analysis. In an attempt to account for this bias, we performed multiple patient‐level and subgroup analyses. Nevertheless, our data reflect real‐world evidence from Germany, suggesting that HIFU is the preferred FT modality.

## Conclusion

In the first epidemiological comparative study on the risk of complications of the most common types of FT for PCa, we demonstrate an overall low risk of perioperative complications. The lowest rate of complications is recorded for the VTP, IRE, and TULSA procedures. This may be attributed to the fact that no whole‐gland treatments are carried out as part of these three modalities. It can therefore be concluded that FT is a safe procedure for localised PCa. Nevertheless, brachytherapy was associated with better perioperative outcomes. However, there is a paucity of long‐term studies on oncological outcomes, as well as comparative studies vs RP, external beam radiotherapy, or other treatment modalities.

## Author Contributions

All authors participated in the drafting, writing, and editing of the manuscript.

## Ethics Statement

Written informed consent from the participants, as well as ethical approval, was not required for the present study in accordance with the national legislation and institutional requirements. All data used in this work are stored anonymised at the German Federal Statistical Office.

## Disclosure of Interests

Maria Apfelbeck has performed the following FT modalities herself in the past and has provided follow‐up care: HIFU and VTP. There are no other benefits or similar to disclose.

## Funding

This research did not receive any specific grant from funding agencies in the public, commercial, or non‐profit sector.
